# Metabolomics Reveals Metabolic Changes Caused by Low-Dose 4-Tert-Octylphenol in Mice Liver

**DOI:** 10.3390/ijerph15122686

**Published:** 2018-11-28

**Authors:** Kun Zhou, Xingwang Ding, Jing Yang, Yanhui Hu, Yun Song, Minjian Chen, Rongli Sun, Tianyu Dong, Bo Xu, Xiumei Han, Keqin Wu, Xiaoling Zhang, Xinru Wang, Yankai Xia

**Affiliations:** 1 State Key Laboratory of Reproductive Medicine, Institute of Toxicology, School of Public Health, Nanjing Medical University, Nanjing 211166, China; zk@njmu.edu.cn (K.Z.); dingxingwang@njmu.edu.cn (X.D.); songyun@njmu.edu.cn (Y.S.); dong317000145@njmu.edu.cn (T.D.); xubo@njmu.edu.cn (B.X.); hanxiumei@njmu.edu.cn (X.H.); keqinwu@njmu.edu.cn (K.W.); xrwang@njmu.edu.cn (X.W.); 2 Key Laboratory of Modern Toxicology of Ministry of Education, School of Public Health, Nanjing Medical University, Nanjing 211166, China; 3 Experiment Center for Teaching and Learning, Shanghai University of Traditional Chinese Medicine, Shanghai 201203, China; jingyang@shutcm.edu.cn; 4 Safety Assessment and Research Center for Drug, Pesticide, and Veterinary Drug of Jiangsu Province, School of Public Health, Nanjing Medical University, Nanjing 211166, China; njjshyh@njmu.edu.cn; 5 Key Laboratory of Environmental Medicine Engineering, Ministry of Education, School of Public Health, Southeast University, Nanjing 210009, China; 101012172@seu.edu.cn; 6 Department of Hygienic Analysis and Detection, Nanjing Medical University, Nanjing 211166, China; zhangxl3@njmu.edu.cn

**Keywords:** metabolomics, 4-tert-octylphenol, low-dose, liver toxicity

## Abstract

*Background*: Humans are constantly exposed to low concentrations of 4-tert-octylphenol (OP). However, studies investigating the effects of low-dose OP on the liver are scarce, and the mechanism of these effects has not been thoroughly elucidated to date. *Methods*: Adult male institute of cancer research (ICR) mice were exposed to low-dose OP (0, 0.01 and 1 μg/kg/day) for 7 consecutive days. Weights of mice were recorded daily during the experiment. Blood serum levels of OP, alanine aminotransferase (ALT) and aspartate aminotransferase (AST) were determined, and haematoxylin-eosin (HE) staining of the liver was performed. We applied an integrated metabolomic and enzyme gene expression analysis to investigate liver metabolic changes, and the gene expression of related metabolic enzymes was determined by real-time PCR and ELISA. *Results*: OP in blood serum was increased after OP exposure, while body weights of mice were unchanged. Liver weight and its organ coefficient were decreased significantly in the OP (1 μg/kg/day) group, but ALT and AST, as well as the HE staining results, were unchanged after OP treatment. The levels of cytidine, uridine, purine and N-acetylglutamine were increased significantly, and the level of vitamin B6 was decreased significantly in mice treated with OP (1 μg/kg/day). The mRNA and protein levels of *Cda* and *Shmt1* were both increased significantly in OP (1 μg/kg/day)-treated mice. *Conclusions*: Through metabolomic analysis, our study firstly found that pyrimidine and purine synthesis were promoted and that N-acetylglutamine was upregulated after low-dose OP treatment, indicating that the treatment disturbed nucleic acid and amino acid metabolism in mice liver.

## 1. Introduction

As a ubiquitous environmental pollutant, 4-tert-octylphenol (OP) is a kind of alkylphenols (APs) with endocrine-disrupting effects. OP has been widely used in the production of industrial and household detergents, and it enters the environment through human use of products containing APs and through the manufacturing waste stream [1]. OP is a kind of lipophilic AP that shows significant bioaccumulation [2,3]. Humans can be exposed to OP through ingestion of contaminated food and drinking water, via inhalation of gases and particles in the air and through the skin. Several studies have shown that OP is easily detected in the environment and in human urine samples [4,5,6]. Evidence indicates that OP is one of the most oestrogenic AP isomers [7], and epidemiological studies have shown that OP exposure is related to adverse reproductive and birth outcomes in humans [8,9,10]. Therefore, environmental levels of OP may be sufficient to disrupt the biological endocrine system and exert toxic effects in humans [10,11].

Previous studies indicated that OP accumulates rapidly in the liver [12], where it exerts its adverse effects [13,14]. It has been reported that OP induces hepatotoxicity in Rana chensinensis and male rats [15,16]. However, the concentrations of OP in these studies were considerably higher than the exposure level in humans. There is a lack of research regarding the effects of low-dose OP on the liver, and the underlying mechanism of these effects remains unclear.

Metabolomics is a rapidly developing approach to detect small molecules in multiple biological samples [17]. The metabolomic approach has become indispensable to system biology [18], which aims to analyse functional changes in different metabolic pathways due to exogenous chemical exposure or diseases. Currently, the analysis of metabolomic profiles is a promising tool which provides novel insights into mechanisms of exogenous chemical toxicity [19,20], including hepatotoxicity [21,22].

In this study, we evaluated the effects of low-dose OP on mouse liver and conducted a hypothesis-free metabolomic analysis to reveal the underlying mechanism.

## 2. Materials and Methods

### 2.1. Chemicals

4-tert-octylphenol (CAS number: 140-66-9, purity ≥ 99.0%) was purchased from Dr. Ehrenstorfer (Augsburg, Germany). All metabolite standards were obtained from Sigma–Aldrich (St. Louis, MO, USA). Methanol and acetonitrile (ACN) were purchased from Merck (Darmstadt, Germany). All chemicals were of chromatogram grade.

### 2.2. Animals and Treatments

Specific pathogen-free (SPF) institute of cancer research (ICR) male mice at 8 weeks of age were purchased from Slaccas (Slaccas Laboratory Animal, Shanghai, China). All mice were housed under controlled temperature (22 °C ± 2 °C) and humidity (40–60%) with a 12 h light/dark cycle and were randomized into 3 groups (*n* = 10 mice/group). The number of mice per group was used based on the “power analysis” (http://3rs-reduction.co.uk) according to a previous report [23]. The animals had free access to food and water and were acclimatized to the laboratory environment for 1 week prior to the start of the experiments. Mice were injected intraperitoneally with saline, 0.01 μg/kg body weight (bw)/day of OP, or 1 μg/kg bw/day of OP daily for 7 consecutive days. The liver has a large blood perfusion, as it is the main metabolic organ, and OP accumulates rapidly [12], meaning that the liver is vulnerable to toxic damage. Therefore, the exposure duration was chosen according to previous studies on hepatic toxicity [24,25]. OP dosages of 0.01 μg/kg bw/day and 1 μg/kg bw/day were chosen according to OP exposure levels in humans [6]. OP solutions were prepared by dissolving the OP standard into saline to the concentrations of 0.001 μg/mL and 0.1 μg/mL. The injection volume per unit bw of mice was 0.1 mL/10g according to the previous studies [26,27]. Body weight and food consumption of all mice were recorded daily. At 7th day after dosing, the mice were fasted for 8 h, anaesthetized and sacrificed, after which blood samples were collected. Then, the livers were dissected and weighed in order to calculate the organ coefficient for each mouse. Liver tissues were stored at −80 °C after snap freezing in liquid nitrogen or fixed in 10% formaldehyde for haematoxylin-eosin (HE) staining. This study was carried out strictly in accordance with the international standards on animal welfare and the guidelines of the Institute for Laboratory Animal Research of Nanjing Medical University. This study was approved by the Animal Ethical and Welfare Committee of Nanjing Medical University (Approval No: IACUC14030184).

### 2.3. Analysis of Serum Levels of ALT and AST

Fresh mice blood samples were centrifuged at 1800× *g* for 15 min at 4 °C to obtain blood serum. Serum ALT and AST are common biochemical markers of liver injury. The serum ALT and AST were analysed using commercially available diagnostic kits (Span Diagnostics, Surat, India) according to a previous method [28].

### 2.4. Histological Evaluations

HE staining was conducted to investigate morphological changes. Pieces of liver from the three groups were fixed in 10% formaldehyde and dehydrated with 70% ethanol. The tissues were embedded in paraffin, and then 5-μm sections were cut and mounted onto slides. The slide sections were stained with haematoxylin and eosin Y. The morphological features of the liver were determined by optical microscopy.

### 2.5. Sample Preparation for the Mass Spectrometry Analysis of Mouse Liver and Blood Serum

Liver sample preparation was performed as follows: 50 mg of frozen liver tissue was shredded by surgical scissors; tissues were mixed with 150 μL ultra-pure water and 600 μL pure methanol. The tissues were ultrasonicated (power: 60%), and the supernatant was obtained after centrifugation (16,000× *g*, 10 min, 4 °C) for dryness. To avoid the heat generated by continuous working probe, ultrasound was on for 6 sec at 78 W and then off for 4 sec per cycle. The total ultrasound generation time was 5 min. After dryness, the residue was reconstituted for metabolomic and targeted S-adenosylmethionine (SAM) analysis. Blood serum sample preparation was performed as follows: 30 μL methanol was added into 10 μL serum. After protein precipitation, the supernatant was obtained after centrifugation (16,000× *g*, 10 min, 4 °C) for dryness. Then, the residue was reconstituted for 4-tert-octylphenol analysis.

### 2.6. Metabolomic Profiling

The metabolomic analysis was done according to the previous report [29]. Briefly, LC-HRMS analysis was performed on a UPLC Ultimate 3000 system (Dionex, Germering, Germany) coupled to a Q-Exactive mass spectrometer (Thermo Fisher Scientific, Bremen, Germany) in both positive and negative modes simultaneously. The heated electrospray ionization (HESI) source was used, and the parameters of the mass spectrometer were set as follows: for the positive mode, a spray voltage of 3.5 kV; for the negative mode, a spray voltage of 2.5 kV; for both modes, a capillary temperature of 300 °C; a sheath gas flow of 50 arbitrary units (AU); an auxiliary gas flow of 13 AU; a sweep gas flow of 0 AU; an S-Lens RF level of 60. Data acquisition was performed in a full-scan mode ranging from 70 *m*/*z* to 1050 *m*/*z*. The instrument was operated at a 70,000 resolution with an automatic gain control (AGC) target of 3 × 10^6^ charges. The UPLC analysis was carried out with a Hypersil GOLD C18 column (100 mm × 2.1 mm, 1.9 μm) (Thermo Fisher Scientific) with column temperature set at 40 °C. A multistep gradient was used with a mobile phase A of 0.1% formic acid in ultra-pure water and mobile phase B of 0.1% formic acid in pure ACN with a flow rate of 0.4 mL/min over a run time of 15 min. The UPLC autosampler temperature was set at 4 °C, and the injection volume for each sample was 10 μL. The MS system was calibrated according to the manufacturer’s instructions. The metabolite identification was based on the comparison of accurate mass and retention time with metabolite standards. All samples were analysed in a randomized fashion to avoid complications related to the injection order.

### 2.7. Targeted S-Adenosylmethionine Analysis in Liver and 4-Tert-Octylphenol in Blood Serum

SAM and OP were detected using UPLC Ultimate 3000 system coupled to a Q-Exactive mass spectrometer. The conditions of the chromatography and mass spectrometer were the same as described in metabolomic profiling. The [M]^+^ ions of SAM at *m*/*z* 399.14451 and the [M − H]^−^ ions of OP at *m*/*z* 205.15979 were monitored.

### 2.8. Real-Time PCR Analysis

Total RNA was homogenized and extracted from snap-frozen liver tissues by use of Trizol (Invitrogen, Carlsbad, CA, USA) according to the manufacturer’s instructions. The concentration of total RNA was determined using a NanoDrop 2000 (Thermo Fisher Scientific, Wilmington, DE, USA). Reverse transcription was performed using a Prime-Script RT Reagent Kit (Takara, Dalian, China) in accordance with the manufacturer’s recommendations. All real-time PCR reactions were carried out on an ABI7900 HT Fast Real-Time System (Applied Biosystems, Foster City, CA, USA) using SYBR Green PCR Master Mix reagent kits (Takara, Dalian, China) according to the manufacturer’s instructions. Specific primers for the genes of interest are listed in Table 1. All oligonucleotide primers were synthesized by Invitrogen (Shanghai, China). All of the PCRs were performed in triplicate, and the specificity of the PCR products was confirmed using melting curve analysis. The 2^−ΔΔ*C*t^ method was used to calculate the relative expression [30]. *Gapdh* was used as an internal control for real-time PCR. The levels of the *Cda* and *Shmt1* genes were normalized relative to the expression levels of *Gapdh*.

### 2.9. Enzyme-Linked Immunosorbent Assay

The protein level of Shmt1 in mice liver tissues was measured using a commercial enzyme-linked immunosorbent assay (ELISA) kit (MyBiosource, San Diego, CA, USA). The protein level of Cda in mice liver tissues was measured using a commercial ELISA kit (LifeSpan Biosciences, Seattle, WA, USA). The data are presented as fold change compared with the mean value of the control group according to the previous report [31]. All procedures were performed in accordance with protocols provided by the kit manufacturers.

### 2.10. Statistical Analysis

Statistical analysis of the data was performed using Stata statistical package (Version 9.2, Stata Corp, LP, Lakeway Drive College Station, TX, USA) and SPSS (version 22.0, SPSS, Inc., Chicago, IL, USA). Statistical comparisons among the three groups were performed by ANOVA followed by Dunnett’s test. The dose effect relationship between OP dosage and weight, metabolite and gene expression data was tested by Spearman correlation test. A *p* value < 0.05 was considered to be statistically significant.

## 3. Results

### 3.1. Changes of Body Weight and Liver Organ Coefficient

**Figure 1:**
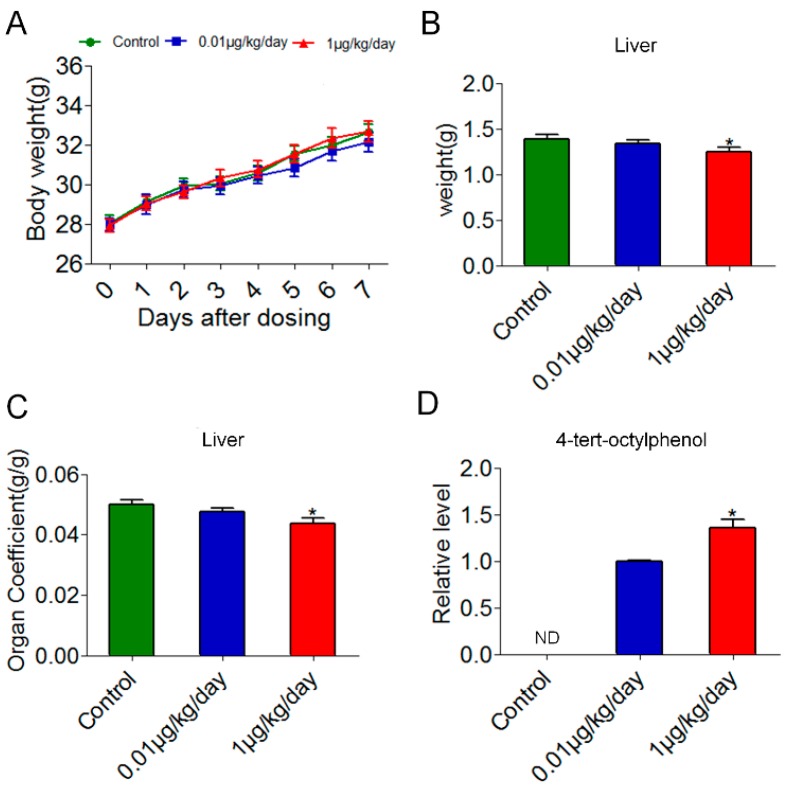
Effects of low-dose 4-tert-octylphenol (OP) on body weight, weight of liver and liver organ coefficient as well as OP blood serum level in mice. (**A**) Body weights of mice were not significantly changed between the control and OP-treated groups. Values are mean ± SEM. (**B**) Weight of liver was decreased significantly in the OP (1 μg/kg/day) group. Values are mean ± SEM. Significance level: * *p* < 0.05 compared with controls. (**C**) Organ coefficient of liver was decreased significantly in the OP (1 μg/kg/day) group. Values are mean ± SEM. Significance level: * *p* < 0.05 compared with controls. (**D**) The blood serum level of OP in the OP (1 μg/kg/day) group was significantly higher than that in the OP (0.01 μg/kg/day) group, and OP was undetectable in the control group. ND: Not detectable. Significance level: * *p* < 0.05 compared with the OP (0.01 μg/kg/day) group using *t*-test.

As shown in Figure 1A, body weight was not significantly affected in the OP-treated mice. Food consumption in the control group, the OP (0.01 μg/kg/day) group and the OP (1 μg/kg/day) group was 5.36 ± 0.59 g/day, 5.14 ± 0.16 g/day and 5.52 ± 0.19 g/day (mean ± SEM), respectively, and there was no significant difference among the groups. Liver weight was decreased significantly in the OP (1 μg/kg/day) group (Figure 1B). The liver organ coefficient was decreased significantly in the OP (1 μg/kg/day) group (Figure 1C) and was negatively correlated with OP doses by Spearman’s correlation test (*r* = −0.458, *p* < 0.05). These results indicated that OP might cause liver injury in relatively low doses, which was dose-related. We detected OP in blood serum and found that OP was undetectable in the control group, while it was detectable in the OP (0.01 μg/kg/day) group and the OP (1 μg/kg/day) group. The level of OP in the OP (1 μg/kg/day) group was significantly higher than that in the OP (0.01 μg/kg/day) group (Figure 1D).

### 3.2. Histological Results

**Figure 2:**
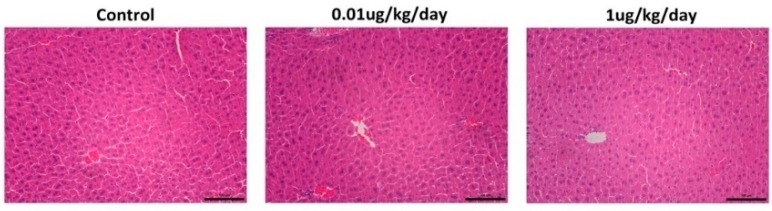
Effects of low-dose OP on liver morphology in mice. Representative images of haematoxylin-eosin (HE) staining in mice liver tissues. No obvious morphological damage was observed in the livers of OP-treated mice. Scale bar = 100 μm.

The results of HE staining showed that there was no obvious morphological change in the liver between OP-treated and control mice (Figure 2).

### 3.3. Results of the Serum ALT and AST

**Figure 3:**
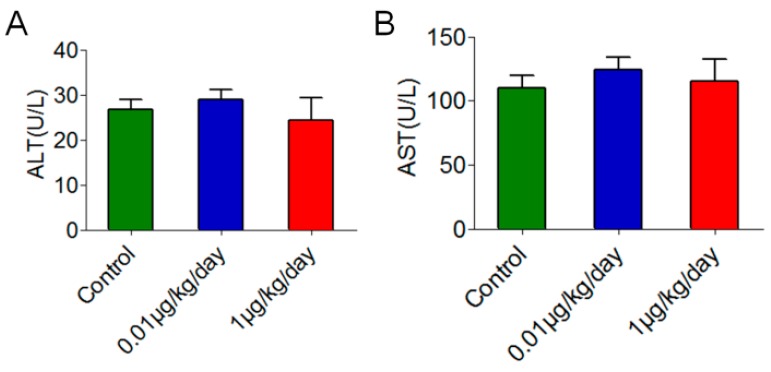
Serum levels of alanine aminotransferase (ALT) and aspartate aminotransferase (AST) in the control and low-dose OP treated mice. (**A**) There was no significant difference in serum levels of ALT in mice after OP treatment. (**B**) There was no significant difference in serum levels of AST in mice after OP treatment.

Figure 3A,B shows that the serum levels of ALT and AST were not significantly changed in the OP (0.01 μg/kg/day) and the OP (1 μg/kg/day) groups compared with those in the control group.

### 3.4. Metabolomic Profiles

A total of 190 metabolites were detected in mouse liver in all groups by LC-HRMS. Among these, uridine, *N*-acetylglutamine, cytidine, pyridoxine, and purine were significantly changed in the liver samples from the OP (1 μg/kg/day) group (*p* < 0.05) (Figure S1). We found that uridine, *N*-acetylglutamine, cytidine, and purine were increased, whereas pyridoxine, also known as vitamin B6, was decreased in mice treated with OP at a dosage of 1 μg/kg/day. Additionally, the relative levels of these metabolites were positively or negatively correlated with OP doses by Spearman’s correlation test in the three groups (*r* > 0.35, for positive correlation; *r* < −0.45, for negative correlation, *p* < 0.05), indicating the dose-related alterations of these metabolites. After classifying the chemicals and mapping the metabolites into general biochemical pathways, as illustrated in the Kyoto Encyclopedia of Genes and Genomes (KEGG) (http://www.genome.jp/kegg/), it became clear that metabolomic disturbances in mouse liver caused by low-dose OP exposure are involved in pyrimidine metabolism (uridine and cytidine), d-glutamine and d-glutamate metabolism (N-acetylglutamine), vitamin B6 metabolism (vitamin B6) and purine metabolism (purine).

### 3.5. Low-Dose OP Promoted Pyrimidine Synthesis through Cytidine Deaminase

**Figure 4:**
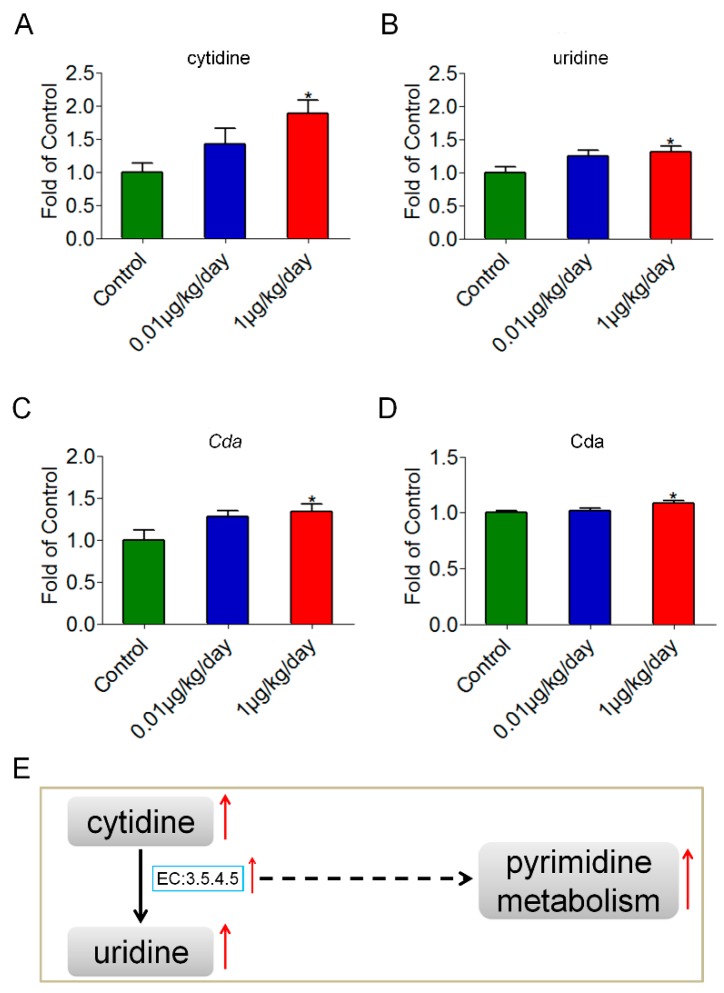
Metabolomic data for pyrimidine metabolism in mouse liver after low-dose OP treatment. (**A**) The relative level of cytidine was increased significantly at the OP dosage of 1 μg/kg/day. (**B**) The relative level of uridine was increased significantly at the OP dosage of 1 μg/kg/day. (**C**) The mRNA level of *Cda* was increased significantly at the OP dosage of 1 μg/kg/day. (**D**) The protein level of Cda was increased significantly at the OP dosage of 1 μg/kg/day. (**E**) Schematic diagram showing that pyrimidine synthesis might be promoted in the livers of mice treated with low-dose OP. Significance level: * *p* < 0.05 compared with controls.

The increased cytidine and uridine were among the most dramatic changes in mouse liver caused by OP treatment at a dosage of 1 μg/kg/day. The relative levels of cytidine and uridine in mice treated with 1 μg/kg/day of OP were significantly higher than those in control mice (*p* < 0.05) (Figure 4A,B). Additionally, the relative levels of cytidine and uridine were positively correlated with OP doses by Spearman’s correlation test in the three groups (*r* = 0.453, *p* < 0.05, for cytidine; *r* = 0.368, *p* < 0.05, for uridine). The mRNA and protein levels of *Cda*, which encodes cytidine deaminase [EC:3.5.4.5] and catalyses the irreversible hydrolytic deamination of cytidine to uridine, were also increased significantly in mice treated with 1 μg/kg/day of OP (Figure 4C,D). The mRNA and protein levels of *Cda* were positively correlated with OP doses in the three groups (*r* = 0.491, *p* < 0.05, for mRNA level; *r* = 0.328, *p* < 0.05, for protein level). All of these results indicated that pyrimidine synthesis might be increased in mouse liver after low-dose OP treatment, which was dose-related (Figure 4E).

### 3.6. Purine Synthesis Was Increased through Pyridoxal-5′-Phosphate (PLP)-Dependent Enzyme-Shmt1 after Low-Dose OP Treatment

**Figure 5:**
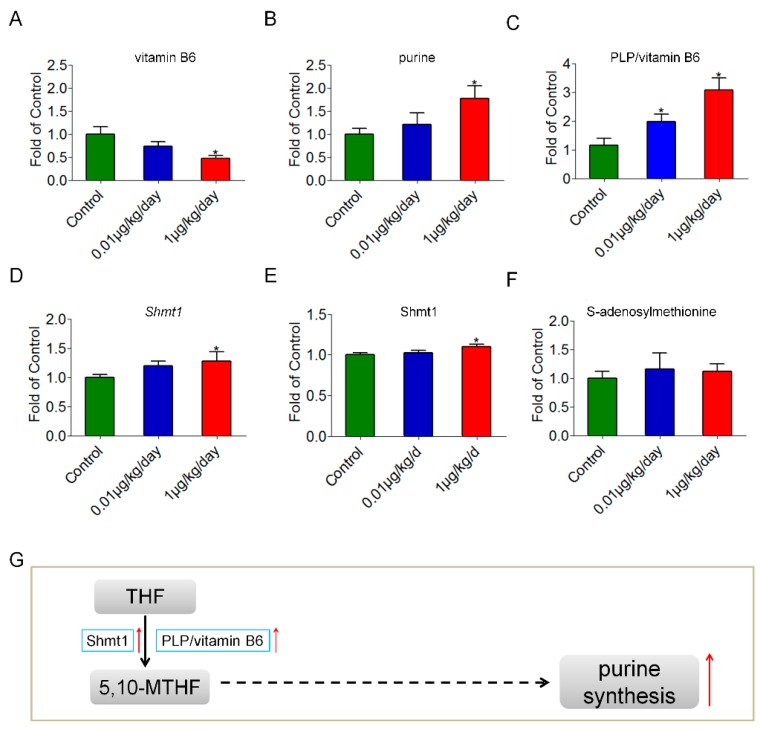
Metabolomic analysis for purine metabolism in mouse liver after low-dose OP treatment. (**A**) The relative level of vitamin B6 was decreased significantly at the OP dosage of 1 μg/kg/day. (**B**) The relative level of purine was increased significantly at the OP dosage of 1 μg/kg/day. (**C**) The Pyridoxal-5′-Phosphate (PLP) relative level to vitamin B6 relative level ratio was increased significantly in the OP treated groups. (**D**) The mRNA level of *Shmt1* was increased significantly at the OP dosage of 1 μg/kg/day. (**E**) The protein level of Shmt1 was increased significantly at the OP dosage of 1 μg/kg/day. (**F**) The relative level of S-adenosylmethionine (SAM) was not significantly changed after OP treatment. (**G**) Schematic diagram showing that the metabolism (the conversion of tetrahydrofolate (THF) into 5,10-methylenetetrahydrofolate (5,10-MTHF)) might be promoted by increasing the expression of Shmt1 (a PLP-dependent enzyme); thus, purine synthesis might be promoted in mice treated with a low dose of OP. Significance level: * *p* < 0.05 compared with controls.

One-carbon metabolism plays a key role in DNA synthesis, DNA methylation, detoxification and protection against oxidation, in which vitamin B6 is a cofactor of several enzymes, including serine hydroxymethyltransferase 1 (Shmt1) [32]. Shmt1 is a PLP-dependent enzyme [33] that catalytically converts serine into glycine. Tetrahydrofolate (THF) receives one-carbon groups from serine and changes into 5,10-methylenetetrahydrofolate (5,10-MTHF), which is involved in purine synthesis [34,35,36]. As shown in Figure 5A, the relative level of vitamin B6 was decreased significantly in mice treated with 1 μg/kg/day of OP. Furthermore, the relative levels of vitamin B6 were negatively correlated with OP doses by Spearman’s correlation test in the three groups (*r* = −0.486, *p* < 0.05). The relative level of purine in mice treated with 1 μg/kg/day of OP was significantly higher than that in the control mice (Figure 5B). Additionally, the relative levels of purine were positively correlated with OP doses by Spearman’s correlation test in the three groups (*r* = 0.437, *p* < 0.05). PLP, the active form of vitamin B6, is an essential coenzyme that is involved in amino acid and nucleotide metabolism [37,38]. PLP was detectable in all mice from the three groups. The PLP relative level to vitamin B6 relative level ratio of each mouse was calculated. Accordingly, this ratio was increased significantly in the low-dose OP treated groups (Figure 5C). Furthermore, the ratios were positively correlated with OP doses by Spearman’s correlation test in the three groups (*r* = 0.670, *p* < 0.05). The mRNA and protein levels of *Shmt1*, which encodes serine hydroxymethyltransferase 1, were increased significantly in the OP (1 μg/kg/day) treated group (Figure 5D,E). Besides, the mRNA and protein levels of *Shmt1* were positively correlated with OP doses by Spearman’s correlation test in the three groups (*r* = 0.234, *p* < 0.05, for mRNA level; *r* = 0.415, *p* < 0.05, for protein level). These results indicated that purine synthesis might be increased in mouse liver after low-dose OP treatment, which was dose-related. This metabolism can further support the formation of SAM, and DNA methylation depends upon the availability of methyl groups from SAM [39,40]. To explore the possible DNA methylation disruption caused by OP from the perspective of metabolites, we detected SAM in the livers and found that it was not significantly changed after low-dose OP treatment (Figure 5F). Thus, OP might not exert an effect on DNA methylation in the liver. The summary of the effect of OP on purine metabolism in the liver is shown in Figure 5G.

### 3.7. Amino Acid Derivative Was Increased after Low-Dose OP Treatment

**Figure 6:**
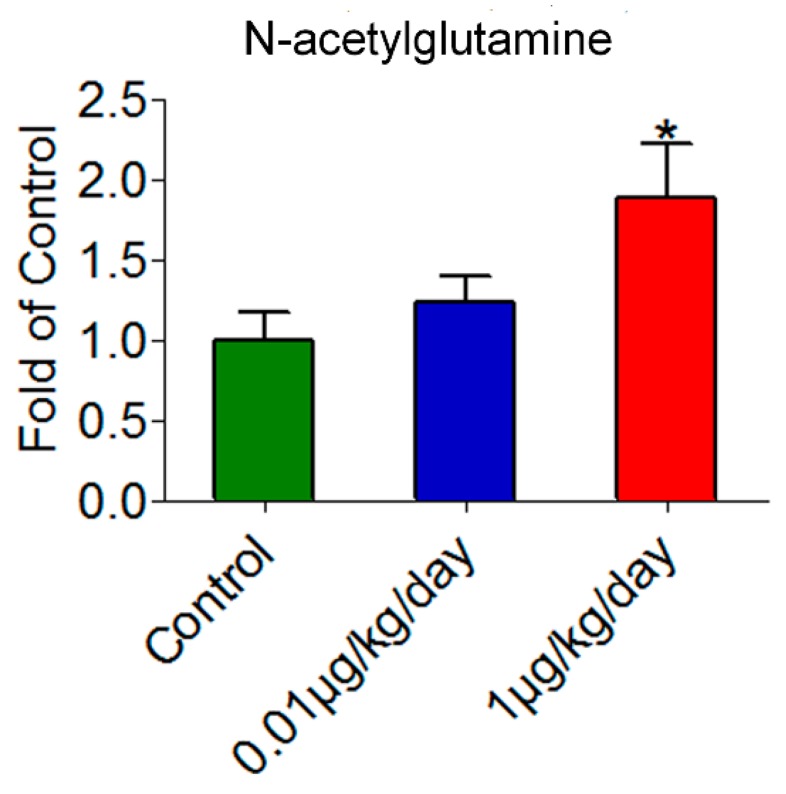
Relative level of N-acetylglutamine in the livers of mice treated with low dosages of OP. The relative level of N-acetylglutamine was increased significantly at the OP dosage of 1 μg/kg/day. Significance level: * *p* < 0.05 compared with controls.

N-acetylglutamine is a kind of amino acid derivative (an acetylated analogue of glutamine) [41]. In this study, compared with the control group, the relative level of N-acetylglutamine was elevated significantly following OP treatment at 1 μg/kg/day (Figure 6). In addition, the relative levels of N-acetylglutamine were positively correlated with OP doses by Spearman’s correlation test in the three groups (*r* = 0.472, *p* < 0.05).

## 4. Discussion

The present study aimed to evaluate the effects of low-dose OP on the male mouse liver and to reveal the underlying mechanism by integrated metabolomic and enzyme gene expression analysis. As a kind of endocrine disrupting chemical, OP has been shown to exert oestrogenic activity in many in vivo and in vitro studies [42,43]. At relatively high doses, OP exerted hepatotoxic effects and induced oxidative stress in male rats [44], indicating that OP has hepatotoxicity. In this study, the results of HE staining showed no obvious morphological changes in the hepatic tissues of low-dose OP treated mice. A previous study has shown that OP at the dosage of 25 mg/kg/day increased the serum levels of ALT and AST and induced liver damage in male Wistar albino rats [44]. In this study, the serum levels of ALT and AST were not significantly changed in low-dose OP-treated mice. However, liver weight and its organ coefficient were decreased significantly after OP treatment at a dosage of 1 μg/kg/day, which indicated that low doses of OP might induce injury in mice liver. Metabolomics is a sensitive tool to detect metabolic dynamics of small molecules in organisms with no obvious damage or diseases caused by exogenous chemical exposure [45,46]. In this study, cytidine, uridine, vitamin B6, purine and N-acetylglutamine were the significantly changed metabolites in the livers of low-dose OP treated mice without obvious liver damage. We found the alterations of these metabolites were dose-related by Spearman’s correlation test among all groups.

The importance of pyrimidines lies in the fact that they are structural components of a broad spectrum of key molecules that are involved in the synthesis of DNA and RNA [47]. Cytidine is the major material for the synthesis of cytosine and uracil. Cytosine hydrolyses to uracil rather rapidly, and cytidine is hydrolysed to uridine at a similar rate [48]. In this study, the levels of cytidine and uridine were increased significantly in mice treated with a 1 μg/kg/day dosage of OP with the increased gene expression of *Cda*, indicating that pyrimidine synthesis was increased in mouse liver after low-dose OP treatment. Uridine, the material basis of RNA synthesis [49], was increased significantly in the OP (1 μg/kg/day) group, which indicated that low-dose OP might affect transcription in the liver. Collectively, pyrimidine metabolism was disturbed in the liver, indicating that DNA and RNA synthesis might be disrupted in hepatocytes after low-dose OP exposure.

PLP is the active form of vitamin B6, which acts as a coenzyme involved in DNA synthesis [50]. In this study, the relative level of vitamin B6 was decreased significantly at the OP dosage of 1 μg/kg/day, and the PLP relative level to vitamin B6 relative level ratio was increased significantly in the low-dose OP-treated mice. These results indicated that low-dose OP might promote coenzyme activation in mouse liver. Shmt1, a PLP-dependent enzyme, catalyses conversion of serine to glycine and therefore connects serine metabolism with glycine metabolism [51]. During this process, THF receives one-carbon groups from serine and changes into 5,10-MTHF, which is involved in purine synthesis. The mRNA and protein levels of *Shmt1* were increased significantly in the OP (1 μg/kg/day) treated group, which indicated that low-dose OP might promote one-carbon unit synthesis by increasing the expression of Shmt1. A previous study has shown that the biosynthetic pathways which employ folate-derived one-carbon units to generate purine [52]. Another study has shown that exogenous chemical exposure induced liver damage in ICR mice through the activation of purine metabolism [53] and that the disturbance of purine metabolism mediated the generation of genotoxicity [54]. In this study, the relative level of purine was increased significantly in the OP (1 μg/kg/day) treated mice, indicating that low-dose OP might promote purine synthesis in the liver and exert hepatotoxic effects.

N-acetylglutamine is an amino acid derivative which is the downstream metabolite involved in d-glutamine and d-glutamate metabolism [55]. Previous studies have shown that urinary N-acetylglutamine could be a potential biomarker to identify several diseases [56,57]. Another metabolomic study showed that liver injury is associated with changes in the metabolism of N-acetylglutamine [58]. In this study, we observed that the relative level of N-acetylglutamine was increased significantly in the OP (1 μg/kg/day) group, indicating that the metabolism of N-acetylglutamine was disturbed in the livers of mice treated with OP at a relatively low dose.

In this study, we only used males to study the metabolomic changes in the liver according to previous reports [59,60]. As a sexually dimorphic effect was previously found in liver treated with another endocrine disrupting chemical, PCB in rats [61], the metabolomic change caused by OP in the liver of females is an interesting topic which needs further investigation with strict control of confounding factors.

## 5. Conclusions

**Figure 7:**
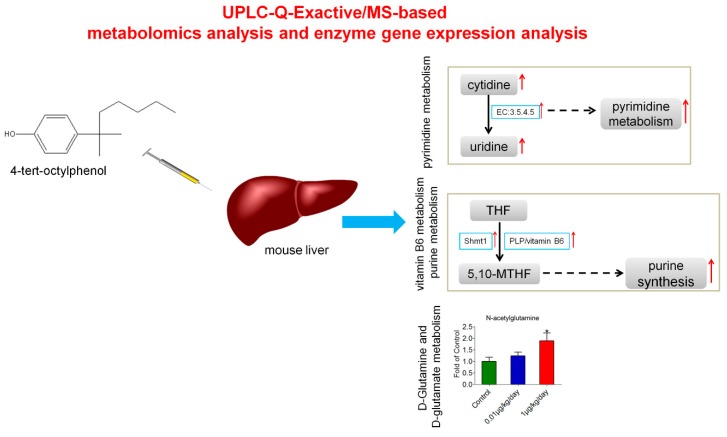
Summary of the integrated metabolomic and enzyme gene expression analysis of mice liver treated with low-dose OP.

By integrated metabolomic and enzyme gene expression analysis, our study firstly revealed that low-dose OP promoted pyrimidine and purine synthesis through the increased expression of Cda and Shmt1, respectively. In addition, N-acetylglutamine was upregulated after low-dose OP treatment. The combination of these results indicated that low-dose OP exposure disturbed nucleic acid and amino acid metabolism in mouse liver, which might exert hepatotoxic effects (Figure 7).

## Figures and Tables

**Table 1:** Primers for real-time PCR.

Target Gene	GenBank Accession no.	Product Length (bp)	Primer Sequences
*Cda*	NM_028176.1	78	Sense: 5′-ATGAGAGAGTTTGGCACCGAC-3′
			Anti-sense:5′-CTCCTGGACCGTCCTGACTA-3′
*Shmt1*	NM_009171.2	94	Sense: 5′-CCCGAAACCAAGTGAACTGGA-3′
			Anti-sense:5′-ACTGGTTCAGAGTTGCCTTGTA-3′
*Gapdh*	NM_001289726.1	124	Sense: 5′-CCCTTAAGAGGGATGCTGCC-3′
			Anti-sense:5′-TACGGCCAAATCCGTTCACA-3′

## Supplementary Materials

The following are available online at http://www.mdpi.com/1660-4601/15/12/2686/s1, Figure S1: The chemical structure of cytidine, uridine, pyridoxine, purine and N-acetylglutamine.
