# Supplementary material for: Metabolomics Reveals Metabolic Changes Caused by Low-Dose 4-Tert-Octylphenol in Mice Liver
Source: Int J Environ Res Public Health. 2018 Nov 28;15(12):2686. doi: 10.3390/ijerph15122686 (PMC6313621; doi:10.3390/ijerph15122686)
Supplement: Supplementary file 1 [file ijerph-15-02686-s001.pdf]

Supplementary Materials

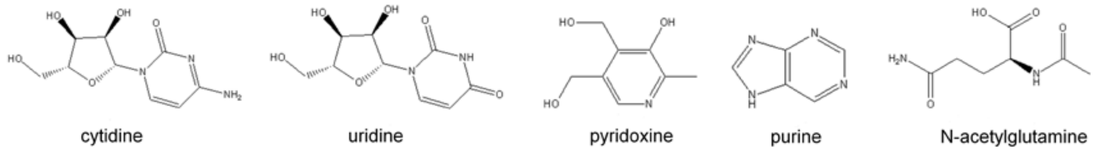

Figure S1. The chemical structure of cytidine, uridine, pyridoxine, purine and N-acetylglutamine.
